# Mapping out market drivers of improved variety seed use: the case of sorghum in Tanzania

**DOI:** 10.1016/j.heliyon.2022.e08715

**Published:** 2022-01-07

**Authors:** Elizabeth P. Kalema, Essegbemon Akpo, Geoffrey Muricho, Justin Ringo, Chris O. Ojiewo, Rajeev K. Varshney

**Affiliations:** a International Crops Research Institute for the Semi-Arid Tropics, Patancheru, 502 324, Telangana, India; b Tanzania Agricultural Research Institute, P.O. Box 1571, Dodoma, Tanzania; c Ecole de Gestion et de Production Végétale et Semencière, Université Nationale d’Agriculture, BP 43, Kétou, Benin; d Formerly of International Crops Research Institute for the Semi-Arid Tropics, Patancheru, 502 324, Telangana, India; e State Agricultural Biotechnology Centre, Centre for Crop Research and Food Innovation, Murdoch University, Murdoch, WA, Australia

**Keywords:** Grain off-takers, Cost and return of sorghum production, Binary logit model, Sorghum improved variety seed

## Abstract

It is understood that the grain market pulls the seed market. The problem of low quality prompted failure of traders and processors to purchase most of the farmers' grain to subsequently drive the use of improved variety seed. The aim of this study is to identify drivers that persuade farmers to use improved variety seeds for grain production. It also assesses factors affecting market participation among small-scale farmers. Descriptive analysis, Binary Logistic model, Probit model and gross margin analysis was conducted from random selected sample of 212 individual farmers, 63 grain off-takers, 3 extension officers and 7 seeds producers through structured interviews. In additional, 80 farmers were interviewed through 10 focus group discussion. The results showed that taste, preferences and price difference between grain and seed were significant and positive drivers that influenced the decision of farmers to use improved varieties at 47% and 0.007%, respectively. Factors such as group membership and farm size were significantly positive affecting farmer's market participation while age was negatively significant affecting farmer's market participation. Gross margin was computed to compare the profit margin between users and non-users of improved variety seeds, where users had high profit margin (530 979.89Tsh/Ha) compared to non-users (472 885.94Tsh/Ha), because non-users incurred high seed cost (54 504.84Tsh/15kg) compared to users of improved variety seeds (39 329.94Tsh/kg). Also, users obtained high grain revenue compared to non-user at 1 353 268.37Tsh and 848 249.11Tsh, respectively. Efforts should be made by value chain actors and other agricultural actors to support farmers based on market demand so they could benefit from high grain quality, quantity and promising grain market.

## 1 Introduction

Sorghum is among the major staple food crops in the world, and it is mostly grown in semi-arid regions. The crop is used as human food and animal feed for forage and fodder, alcoholic beverages, and biofuels (Prasad and Staggenborg, 2011: Orr et al., 2020). Quantitatively, it is the world's fifth important cereal crop in production after wheat, rice, maize, and barley (FAOSTAT, 2019). Recent statistics show that about 57 million tons were produced worldwide, and USA is the leading producer (FAO, 2017).

Africa has a mean yield of 0.8 t/ha from the cultivated area of 24 million hectares with 20 million tons per year (FAOSTAT, 2015) that makes sorghum the second most important cereal grain in Africa after maize (Msongaleli et al., 2017). In Tanzania, sorghum is the third most widely grown cereal after maize and rice with a total of 834,284 ha which was planted with a total production of 500,000 tons (FAOSTAT, 2018), whereas the average acreage at national level per household was 0.67 ha in 2017 (Tshibaka, 1989).

In the country, sorghum is mostly grown in semi-arid regions of Dodoma, Singida, Mara, Shinyanga, Mwanza and Tabora. Sorghum is mostly planted in central zone which produce 42.98% of the sorghum while the least producing zone is coastal zone which produce 4.15% of the sorghum in the country (URT, 2017). Based on URT (2017) in Tanzania, sorghum has been mostly used for consumption rather than commercialization, where 90.4% of sorghum grain was used for consumption and 9.6% was used for commercialization at national level (URT, 2017). The crop is critical for national food and nutrition security besides being an important source of cash income for households that grow it as a cash crop. In Tanzania, sorghum is mainly used as a human food, highly demanded as a beer ingredient by Tanzania Breweries Company Limited (TBL) and Serengeti breweries Limited (SBL) and local brewers. Furthermore, its stovers are used to feed animals (poultry feed industries), flour making that is highly demanded by sick people mainly diabetic and children, also it used as wood fuel and light fencing materials around homesteads (Kidanka, 2016). There have been inconsistence trend of acreage and production over the years (FAOSTAT, 2019).

In 2018 commercialization level in Tanzania increased where 17% of the sorghum production was traded (FEWS NET, 2018) but still there is an inconsistent trend in farmers’ market participation (URT, 2017). Farmers in Africa and elsewhere participate in the market since it increases household income, gives off-farm employment (Braun et al., 1991) and they realize their comparative advantage in agriculture farming activities and specialization (Wickramansinghe et al., 2014).

Due to the importance of the sorghum in the country, farmers are highly supported and advised by stakeholders to use sorghum improved varieties and to follow best agronomic practices to improve yields, profitability and enhance livelihood (Kaliba et al., 2017). Grain off-takers mainly aim for profit making out of grain produced by farmers, but it is hard for them since farmers produce does not meet the market demand mostly in terms of quality needed by processors and consumers.

Despite efforts and recognized improvement made by the public, private and development institutions, the use of improved varieties and commercial farming of sorghum is still low in the country. And when farmers manage to market their grain, the off-takers offer them low price. This does not encourage farmers to use improved sorghum variety. Thus, most of the farmers use their own-saved seeds. Therefore, The aim of the study is to understand what motivates farmers to go for improved sorghum varieties by identifying the drivers that persuade farmers to grow sorghum improved varieties, to assess factors affecting market participation among small-scale sorghum farmers and the comparative advantage of using improved varieties.

## 2 Theoretical framework

This paper used three classes of models to analyze the main drivers for small-scale farmers to go for improved sorghum variety and to assess the factors affecting market participation among small-scale farmers. The first model involves the innovation-diffusion model or transfer of technology (Rogers, 2003). In this model, knowledge is transferred from its source to sorghum small-scale farmers through extension system and its diffusion depends on personal characteristics of the potential user. It is assumed that the technology is appropriate for use unless it is hindered by lack of effective communication (Negatu and Parikh, 1999). The second lens of analysis involves technological characteristics. According to Scoones and Thomson (1994), the decision to use and diffusion process is vital and based on agro-ecological, socio-economic, and institutional context. It also depends on perceptions of the potential user (Gould et al., 1989). It implies that the early involvement of farmers in technological development process is particularly important to determine the probability of adoption.

The third theory is profit and utility maximization theory. Farmers are considered to be rational in decision making with resource constraints, thus choosing alternatives that maximizes utility (McFadden, 1973). In this case, adopting productivity enhancing technologies example improved seeds and participating in grain market if the perceived benefits by farmers to be obtained from its turns out positive, then sorghum farmers would go for these alternatives. Pandey (2016) asserts farmers decisions are influenced by perceived benefits, opportunities and constraints. Sorghum farmers perceived benefits in adoption of improved seeds and markets analyzed include increased profitability, income, food security, reduced poverty as some of the expected outcomes, although this study has considered to analyze profitability as the study's scope. From profit and utility maximation theory perspective, most of econometric models used are binary ones (i.e probit and logit model) since adoption and market participation decisions are dichotomous choice (yes = 1/no = 0). Furthermore, gross margin analysis was also used as a proxy for profitability analysis in this study (Rao et al.,2017: Engwali et al.,2019) to show the profitability of using improved varieties (Kimbi et al., 2020).

Binary Logistic model was used to analyze market factors that drive small-scale sorghum farmers in using improved sorghum variety. Mutanyagwa et al. (2018) also used binary logistic model to determine market factors that motivate farmers' choice on the use of improved sorghum variety. Probit model was used to assess factors affecting market participation among small-scale farmers. Mbitsemunda and Karangwa. (2017) confirmed that age, sex, group membership, seed accessibility, off farm income, credit accessibility, number of years in school and farm size influence farmers’ decision to use sorghum improved variety. Moreover, it is conceptualized that profitability of sorghum improved grain may directly be linked to these market factors and socio-economic ones.

## 3 Methodology

### 3.1 Study area and data collection

The study was conducted in ten districts of which eight had undergone Hope II and AVISA project interventions through facilitated seed access to farmers. The eight intervention districts were Mkalama, Singida DC, Iramba, Ikungi, Serengeti, Rombo, Momba, and Nkasi while the two non-intervention districts were Kongwa and Tarime. Sorghum grains off-takers regions selected for survey were in Dodoma, Singida, Dar es Salaam, Arusha, Songwe, Mbeya and Kilimanjaro. These grain off-takers were interviewed at marketplaces, company headquarters and warehouses. A four-stage stratified sampling technique was employed to select the study site with the consideration of the importance of sorghum production and implementation of the sorghum dissemination project. Administrative zone was the first stratum, followed by regions which were Dodoma, Singida, Kilimanjaro, Songwe, Mara and Rukwa. Then, districts were selected as the third stratum followed by villages as the forth stratum. Primary data was collected using key informant interviews, focus group discussions (FGDs) and a structured questionnaire from individual farmers and grain-off takers. A total of 212 individual farmers, 80 farmers from 10 focus groups were randomly selected, 63 grain off-takers, 3 extension officers and 7 seed producers from 10 districts. The sample frame of 450 sorghum farmers from 18 villages were obtained with the help of village executives and extension officers. Even if our study did not include the use of human or animal samples or experiments, we sought necessary ethical clearance from the Institutional Ethics Committee (IEC) of ICRISAT. For all interviewees, informed consent was first obtained before proceeding to the interviews and data were anonymously collected.

### 3.2 Data analysis

We analyzed data with SPSS 20 and STATA 13 through descriptive statistics, binary logistic model, probit model and gross margin analysis. We used descriptive statistics to characterize respondents, to determine grain market demand and sorghum quality attributes (product type) in relation to demand preferences inside and outside the country.

#### 3.2.1 Drivers that influenced farmers’ use of improved sorghum varieties

Binary logistic model was used to assess drivers of farmers’ use of improved sorghum varieties, as it resolves problem of heteroscedasticity (Greene, 2008). The variables include sorghum grain price, taste and preferences, targeted consumers and price difference between grain and seed. The probability (Pi) for farmer to adopt improved variety of sorghum is presented in Eq. (1).

(1) $$Z_{i}=\beta_{o}+\overset{n}{\underset{i=1}{\sum }}\beta_{1}X_{i}$$

where β_o_ is constant and Z_i_ is 1 when improved variety is chosen; Z_i_ is 0 when otherwise (equation 1). Eq. (1) represents a binary choice model estimating the probability of choosing a given technology (Z) as a function of independent variables (X).

(2) $$Prob\left(Z=1\right)=\left(\beta^{\prime }X_{i}\right)$$

(3) $$\left(Z=0\right)=\left(1-\beta^{\prime }X_{i}\right)$$

with Z_i_ representing the observed response for i^th^ observation of the response variable Z. Zi = 1 means a user or farmers who use improved varieties of sorghum (equation 2) and Zi = 0 mean a non-user or farmers not using improved varieties of sorghum (equation 3). X_i_ represent a set of independent variables like price of sorghum grain, taste and preferences, targeted consumers and price difference between grain and seed associated with the i^th^ respondent farmer, which determine the probability of using a certain variety (P). The function may take the form of a normal, logistic or probability function. The Binary logit model uses a logistic cumulative distributive function to estimate Z.

(4) $$P\left(Y=\frac{1}{X}\right)=\frac{e^{z}}{1+e^{z}}$$

(5) $$P\left(Y=\frac{0}{x}\right)=1-\frac{e^{z}}{1+e^{z}}$$

(6) $$Z=\beta_{i}X_{1}+\beta_{2}X_{2}+\ldots +\beta_{k}X=\overset{K}{\underset{i=1}{\sum }}\beta_{i}X_{i}$$

where, k represented number of independent variables to be analyzed in the study.

The empirical model for the binary logit model estimation is specified in Eq. (7).

(7) $$z=Ln\left(\frac{Pi}{1-pi}\right)=\beta_{o}+\beta_{1}X_{1}+\beta_{2}X_{2}+\beta_{3}X_{3}+\beta_{4}X_{4}+\ldots +\varepsilon$$

where $Ln\left(\frac{Pi}{1-pi}\right)$ in the formula represents log-odds in favor of farmer's decision to use improved variety seeds or not (equation 7). It is logarithm of the ratio of probability of choosing certain improved variety (p) to probability of doing otherwise.

(1-p). The ratio shows the odds ratio of probability of using improved variety seeds to not use it. That means it is the ratio of probability of using improved sorghum variety (p) to not using it (1-p).

Specific variables to include in the model are described in Table 1.

**Table 1:** Description of variables in binary logistic model analysis

Variables	Variable type	Description	Expected signs
Dependent variable			
The use of improved sorghum variety	Dummy	1 if the farmer uses improved sorghum variety, 0 if otherwise	None

Independent Variables			
Price of sorghum grain	Continuous	Sorghum grain price (Tsh)	+/-
Taste and preference	Dummy	1 if grain is from improved variety seed, 0 if otherwise	+
Target consumers	Dummy	1 if Middlemen, traders, individual consumer and institutional,0 otherwise	+
Price between grain and seeds	Continuous	Differences in price between grain and seeds (Tsh)	+

#### 3.2.2 Gross margin analysis

Gross margin (GM) analysis was conducted to compare profit margin for users and non-users of improved sorghum variety. GM is computed as total revenue (TR) less total variable costs (TVC). TR characterize the amount of grain harvested for each farmer and price unit offered to the grain among users and non-users of improved sorghum variety (equation 8). Also, we test the hypothesis there is no significant difference in gross margin of farmer using and not using improved variety of sorghum. And it was assumed that the fixed costs are negligible, hardly affecting the viability of enterprise such as cost of acquiring land, farming equipment.

(8) $$\begin{array}{l} {\text{GM}}_{\text{i}}=\sum {\text{TR}}_{\text{i}}-\sum {\text{TVC}}_{\text{i}}\;\text{i})\\ \text{GM}={\text{Q}}_{\text{j}}{\text{P}}_{\text{i}}-{\text{X}}_{\text{i}}{\text{P}}_{\text{xi}}\;\text{ii}) \end{array}$$

where:

GM_i_ – Gross Margin (TSh/ha) of i^th^, users/non-users, ƩTR_i_ - total revenue from sales of i^th^.ƩTVC_i_-total variable costs spent on one ha during i_th_ production. Q_j_-output, P_i_-price of output produced X_i_-input P_xi_-cost of input.

Subsequently, a t-test was conducted to compare GM of user and non-users of improved sorghum varieties (Eqs. (9) and (10)).

(9) $$t=\frac{M_{x}-M_{y}}{\sqrt{\frac{S_{X}^{2}}{n_{x}}+\frac{S_{Y}^{2}}{n_{y}}}}$$

where, M_X_ = Mean of user, M_y_ = Mean of non-users, S_x_ = Standard deviation of users,

S_y_ = Standard deviation of non-users, n_x_ = Total number of users, n_y_ = Total number of non-users.

(10) $$S^{2}=\frac{\sum {\left(x-m\right)}^{2}}{n-1}$$

where, *x* = individual values, *M* = mean, n = total number of farmers (user or non-users).

#### 3.2.3 Small-scale sorghum farmer's choice to participate in market

Probit model was used to analyze factors affecting market participation decision of sorghum small-scale farmers in Tanzania. Small-scale farmers’ decision to participate in the market is influenced by many socio-economic factors (Gebremedhin and Jaleta, 2010b). Explanatory variables used include age, sex of the household head, group membership, seed accessibility, number of years in school, off farm income, credit accessibility and farm size. Probit model was employed mainly because of its assumption of normal probability distribution (Wooldridge, 2010). It also compels the disturbance term to be homoscedastic (Domenlich and McFadden, 1975).

The relationship between market participation decision and the factors affecting the decision can be expressed as in Eq. (11).

(11) $$P_{i}=P_{i}\left(Y_{i}=1\right)=Q\left(X_{i},e\right)\left(i=1,2,3\ldots .n\right)$$

The model assumes that the probability of *i*^th^ farmer participating in the market Pi (Yi = 1) is a function of explanatory variables (*X*_i_) shown the unknown parameter vector. The functional specification is presented in Eq. (12).

(12) $$\text{Participation ​in ​market}=\beta_{0}+\beta_{1}X_{1}+\beta_{2}X_{2}+\beta_{3}X_{3}+\ldots +\beta_{i}X_{i}+e_{i}$$

where $\beta_{0}$....... $\beta_{i}$ = coefficient of the explanatory variables and $e_{i}$ = the disturbance term.

Specific variables to include in the model are described in Table 2.

**Table 2:** Description of variables in probit model analysis.

Variables	Variable type	Description	Expected signs
Dependent variable			
Participation	Dummy	1 if the farmer participates,0 if otherwise	None

Independent Variables			
Age	Continuous	Age of household head in years	+/-
Sex	Dummy	1 if farmer is male, 0 otherwise	+
Seed accessibility	Dummy	1 if farmers had access to seed,0 otherwise	+
Group membership	Dummy	1 if a member of cooperative,0 otherwise	+
Years in school	Continuous	Number of years in school	+
Off farm income	Dummy	1 if farmers had off farm income,0 otherwise	+/-
Farm size	Continuous	Total farm size (ha)	+
Credit accessibility	Dummy	1 if farmers had access to credit.0 otherwise	+

## 4 Results

### 4.1 Social economic characteristics of the respondents

#### 4.1.1 Social economic characteristic of small-scale sorghum farmers

Based on sampled sorghum farmers, descriptive analysis shows that male respondents were greater than female respondents where male respondent accounted for 61.8%. About 54.2% of the respondents were 41–60 years old, followed by 19–40 years old (32.1%), above 61 years old were 13.2% and below 18 years old were 0.5%. On the other hand, 82.1% of respondents spent 1–7 years in school, and 11.8% of respondents did not attend school. As indicated in Table 3, more than three quarters of the respondents (77.7%) owned below 1ha of land, 20.3% of respondent owned 1–3 ha and 2.0% of respondents owned above 3 ha. Further, the study shows that 60.4 % of sampled farmers were in farmer group. Also 56.6% of respondent's engage in sorghum production for subsistence purpose while 40.6% produce for both subsistence and commercial purposes and 2.8% grow sorghum for commercial purpose. About 93.9% of sampled farmers did not have access to the market. Also, around 78.3% of the sampled farmers did not have access to improved sorghum varieties.

**Table 3:** Socio-economic characterization of sorghum small-scale farmers in Tanzania (n = 212).

Household variables	Categories	Percent responses (%)
Male respondents	1 = Yes; 0 = Otherwise	61.8
Age	0–18 years	0.5
	19–40 years	32.1
	41–60 years	54.2
	61 years and above	13.2
Number of years in school	0	11.8
	1–7	82.1
	8–11	3.3
	>12	2.8
Farm size (ha)	<1	77.7
	1–3	20.3
	Above 3	2.0
Belongs to farmer group	1 = Yes; 0 = Otherwise	60.4
Main purpose of producing sorghum	Subsistence	56.6
	Commercial	2.8
	Both subsistence and commercial	40.6
Accessible to markets	1 = Yes; 0 = Otherwise	6.1
Accessible to improved sorghum variety	1 = Yes; 0 = Otherwise	21.7

#### 4.1.2 Social economic characteristics of grain off-takers

Sorghum trading was dominated by male respondents (84.1%) compared to female respondents (Table 4). More than half of respondent (56.6%) were aged between 41-60 years old, followed by 39.7% who were aged between 19-40 years old. Around 65.1% of respondent spent 1–7 years in school, followed by 19.1% of respondent who spent 8–11 years in school, and 19.1% spent more than 12 years in school. About 33.3% of sampled grain off-takers transact below 50 tons per year, followed by 31.7% of sampled grain off-takers who trade over 500 tons per year of sorghum grain, 22.2% of respondent traded 50–250 tons per year of sorghum grain and 12.8% traded 251–500 tons per year of sorghum grain. And 14.3% of sampled grain off-takers belonged in a farmers group.

**Table 4:** Social economic characteristics of grain off-takers.

Variables	Categories	Percentages (%)
Male respondent	1 = Yes; 0 = Otherwise	84.1
Age	<18	-
	19–40	39.7
	41–60	56.6
	>60	3.7
Number of years in school	0	6.3
	1–7	65.1
	8–11	19.1
	>12	9.5
Capacity (tones)	<50	33.3
	50–250	22.2
	251–500	12.8
	>500	31.7
Group belong	1 = Yes; 0 = Otherwise	14.3

### 4.2 Market demand drivers of improved variety use by farmers

Binary logit model results suggested that taste and preference have positive influence on adoption of improved sorghum varieties. Whereas taste and preference of sorghum grain increase, the probability of farmers to use improved sorghum variety seed increases by 47%. Also, price difference between grain and seed has positive effect on the use of improved variety seeds. The higher the difference in seed and grain price (price of grain being higher than that of seed), the higher the probability of farmers to adopt improved sorghum variety about 0.007% (Table 5).

**Table 5:** Estimated coefficient and marginal effect of binary logit model.

Variables	Coefficient	Std. err	P|z|	Marginal effect
Sorghum grain price (Tshs)	0.00023	0.00112	0.841	
Taste and preference (1 = grain from improved variety seeds)	3.15086	0.45964	0.000	(0.47)∗∗∗
Targeted consumers (1 = Middlemen, traders, individual consumer, institutional)	-0.40272	0.60178	0.503	
Price different between grain and seeds (Tshs)	0.00050	0.00016	0.001	(0.00007)∗∗∗

∗∗∗Significant at 1%, ∗∗ Significant at 5%, ∗Significant at 10%.

Log livelihood -99.040697.

Prob > chi^2^ 0.0000.

Pseudo R^2^ 0.3021.

Note: R^2:^ regression, chi^2^: chi square.

### 4.3 Factors affecting market participation decision among small-scale sorghum farmers

The likelihood ratio statistic as indicated by chi-square is highly significant (p < 0.0000) suggesting that the model had strong explanatory power (Table 6). The explanatory variables farm size and group membership have positively and significantly affected the farmers' decision to participate in the market. Marginal effect revealed one unit increase in farm size and group membership, increases the probability of farmers' market participation by 2.4% and 16.1%, respectively. However, age has inverse relationship with farmers’ decision to participate in the market, whereas as farmers age increase the probability of farmer to participate in the market decrease by 0.8%

**Table 6:** Probit model analysis for factors affecting market participation among small-scale sorghum farmers.

Probit mode	Robust			Marginal effect		
Variable	Coef.	Std.Err	P>|z|	dy/dx	Std.Err	P>|z|
Age	-0.023∗	0.008	0.003	-0.008	0.003	0.001
Sex	-0.163	0.191	0.396			
Seed accessibility	-0.191	0.229	0.405			
Group membership	0.452∗∗	0.198	0.023	0.161	0.067	0.018
Years in school	0.003	0.038	0.931			
Off farm income	0.101	0.185	0.587			
Farm size	0.067∗∗∗	0.039	0.084	0.024	0.013	0.076
Credit accessibility	-0.408	1.264	0.747			
LR chi2 (8)	16.57					
Prob > chi^2^	0.0349					
Pseudo R^2^	0.0596					
Log likelihood	-130.76934					

∗∗∗ 1% significant level ∗∗ 5% significant level ∗ 10% significant level.

Note: R^2^: regression, LR: Linear regression, chi^2^: chi square.

### 4.4 Gross margin for users and non-users of improved sorghum varieties

Gross margin analysis shows that users had high production cost (530,979.89Tsh/ha) compared to non-users (472,885.94 Tsh/ha). Sampled farmers who used improved variety seeds incurred high production cost due to high cost in land acquisition (268,313.95 Tsh/ha), fertilizer cost (17,718.02 Tsh/ha), weeding cost (935,46.51Tsh/ha), ridging cost (8,575.58 Tsh/ha), insecticides cost (9,898.26Tsh/ha) and transportation cost (27,848.84 Tsh/ha) compared to non-users of improved variety seeds. But Non-users incurred high cost in seed acquisition (54,504.84T Tsh/ha), and other cost like, harvesting cost (8,412.79 Tsh/ha), pesticides cost (12,500T Tsh/ha), security cost (17,461.243 Tsh/ha), grading cost (10,218.02 Tsh/ha) and packaging cost (15,294.89Tsh/ha) compared to users who incurred 39,329.94 Tsh/ha in seed acquisition. Also, users have high revenue compared to non-user due to high price of their grain (424.78Tshs/Kg) and high yield obtained (3 185.81 kg/ha) from the use of improved sorghum variety. Therefore, users had high gross margins compared to non-user with mean of 8.296 Tsh and 3.687 Tsh respectively as shown in Table 7.

**Table 7:** Gross margin for user and non-user of improved variety seeds.

Variables	Users		Non-users	
	Units/ha	Amount (Tshs/Unit)	Units/ha	Amount (Tshs/Unit)
A: Gross revenue				
Average yield (Kg)	3185.81		2163.68	
Average price (Tshs/kg)	424.78		392.04	
Total revenue		1353268.37		848249.11
B: Variable Cost				
Land	1 ha	268313.95	1 ha	230188.95
Seeds	15 kg/ha	39329.94	15 kg/ha	54504.84
Fertilizer	1 ha	17718.02	1 ha	5687.98
Weeding	1 ha	93546.51	1 ha	61937.98
Ridge	1 ha	8575.58	1 ha	2228.68
Insecticides	1 ha	9898.26	1 ha	2029.07
Pesticides	1 ha	1482.55	1 ha	12500
Threshing and winnowing	1 ha	24139.53	1 ha	27074.61
Harvesting	1 ha	4229.65	1 ha	8412.79
Transport	1 ha	27848.84	1 ha	26346.89
Security	1 ha	14651.16	1 ha	17461.243
Grading	1 ha	6468.02	1 ha	10218.02
Packaging	1 ha	14777.81	1 ha	15294.89
Total Variable Cost		530979.89		473885.94

C: Gross margin per acre = Gross income -Total variable cost		1353268.37		374363.17

Mean	1 ha	8.296	1 ha	3.687
t-test		-2.272		
P (T<=t)		0.024∗		

∗Significant at 0.05.

Note:1USD = 2,283Tshs

### 4.5 Other attributes that led to the use of improved sorghum varieties

#### 4.5.1 Main grain off-takers, quality demand and market drivers for each product type

The major sampled grain off-takers were traders (93.6%) that accounted for both small and larger traders. Traders operating inside the country accounted for 71.1%, whereby Central zone traded 241,153tons/year, Southern Highlands traded 71,350tons/year, Northern Highlands traded 23,860tons/year and Costal zone traded 7,869 tons/years. While 23.8% of the traders traded both in and outside the country and about 5.1% exported grain to neighboring countries like Rwanda, Burundi, and Kenya.

**Figure 1:**
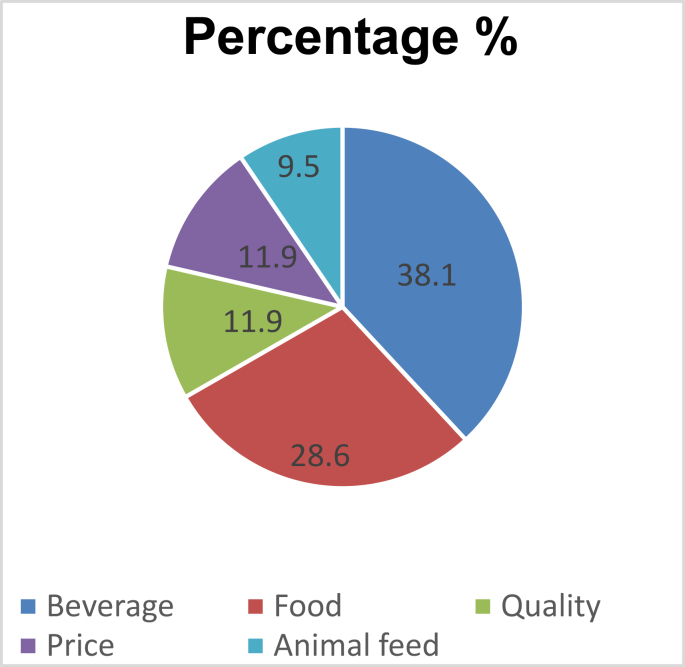
Market drivers for sorghum product type.

For grain consumption, market was driven by the uses of grain, quality, and price. Consumers preferred white varieties (local long white sorghum and the improved white ones) as well as red varieties since it is good for flour, porridge, and local brewery. Major driver of consumers to purchase sorghum grain were beverage (38.1%) and food (28.6%) as shown in Figure 1.

#### 4.5.2 Main uses of sorghum in Tanzania

The 90.4% of sorghum produced was used for consumption and 9.6% were traded at national level. Central zone traded more sorghum (42.81%) compared to other zones, followed Lake zone (28.07%), Western zone (13.11%), Northern Highlands (9.31%), Southern Highlands (6.55%), Southern zone (0.15%) and Coastal zone being least traded sorghum zone (0%). Region wise, Dodoma region led sorghum trade by 3.18%, followed by Tabora region (1.14%) and Morogoro and Pwani region did not engage in sorghum trade (Table 8).

**Table 8:** Main use of sorghum in Tanzania.

Zone	Region	Production (tons)	Consumption (tons)	Commercial (tons)
Southern	Mtwara	3847	3847 (0.83)	0
	Lindi	21497	21432 (4.62)	65 (0.01)
Southern Highlands	Mbeya	28931	26601 (5.73)	2330 (0.50)
	Iringa	1665	1521 (0.33)	144 (0.03)
	Rukwa	3177	2817 (0.61)	360 (0.08)
	Ruvuma	34	26 (0.01)	8 (0.001)
	Katavi	4033	3958 (0.85)	75 (0.02)
Central	Dodoma	133976	119119 (25.68)	14777 (3.18)
	Singida	65533	61230 (13.19)	4303 (0.93)
Coastal zone	Morogoro	7508	7508 (1.62)	-
	Pwani	11759	11759 (2.53)	-
Northern Highlands	Arusha	957	892 (0.19)	65 (0.01)
	Kilimanjaro	86	23 (0.001)	63 (0.01)
	Manyara	13412	9389 (2.02)	4023 (0.87)
Lake	Mwanza	15464	13661 (2.94)	1803 (0.39)
	Kagera	1780	1308 (0.28)	472 (0.10)
	Geita	14911	12240 (2.64)	2671 (0.58)
	Shinyanga	29325	26950 (5.81)	2375 (0.51)
	Simiyu	42168	41598 (8.96)	570 (0.12)
	Mara	26603	21983 (4.74)	4620 (1.00)
Western	Kigoma	1225	689 (0.15)	536 (0.12)
	Tabora	36358	310526.69)	5306 (1.14)
Grand-total		464249	419603 (90.4)	44566 (9.6)

Note: values in parenthesis are percentages of main use of sorghum.

#### 4.5.3 Sorghum quality attributes (product type) in relation to demand preferences inside and outside the country

The white varieties are highly demanded compared to red and tan varieties. Geographically, white sorghum varieties were highly demanded in Southern Highlands, Central Zone, Coastal Zone and Northern Highlands. On the other hand, red varieties were highly demanded in the Lake Zone, Northern Highlands, Coastal Zone, and Central Zone and while tan-coloured varieties were only demanded in the Lake Zone. Outside the country, white-coloured varieties were mostly preferred in Kenya and Uganda, while red-coloured varieties were demanded in Burundi and Rwanda. Tan coloured varieties were preferred in Uganda, Kenya and Rwanda as shown in Table 9. A total 5.1% of sorghum produced in Tanzania is exported to the neighboring countries. For exported sorghum, Uganda and Rwanda were the leading importing countries, accounting for 53.6% and 45.6%, respectively. Other countries were Kenya (0.5%), Burundi (0.2%) and UAE (0.1%).

**Table 9:** Sorghum grain colour preferred by grain off-takers in different markets.

Country	Zones	White (%)	Red (%)	Tan (%)
Tanzania	Central	97.2	2.8	0.0
	Coastal	84.0	16.0	0.0
	Northern Highlands	66.5	33.5	0.0
	Southern Highlands	100.0	0.0	0.0
	Lake	9.0	81.7	9.3
Kenya		86.0	0.0	14.0
Burundi		0.0	100	0.0
Rwanda		18.2	72.7	9.1
Uganda		80.0	0.0	20.0

## 5 Discussion

### 5.1 Market demand drivers on the use of improved varieties

In developing countries most farmers produce what they have and not what is demanded in the market. Grain off-takers offer prices to farmers subject to the grain demand and supply forces in the market. Market demands quality grains, and it is up to farmers to supply what is demanded in the market. Failure to supply what is needed in the market gives grain off-takers hard time to engage in the market and this results into low price to farmer's grains. For farmers to obtain better prices, their sorghum grain should be subjected to market demand in terms of quality and quantity. Through the use of improved seed variety, sorghum farmers are guaranteed to meet market demand (Guei et al., 2011). One of the drivers which positively influence farmer's decisions on the use improved sorghum variety was taste and preference. Grain with good quality and taste is highly preferred and demanded in the market, where farmers had the guarantee of selling their grains and being offered high price that will result to high revenue. Brewing companies tend to offer better price for sorghum through this is only possible if the produce meets their requirements in terms of quality, taste, texture, moisture, and color. For example, Serengeti Breweries Limited (SBL) increased their demand of sorghum for breweries (American sorghum, 2016). With the increase in grain quantity demand by breweries companies, farmers have the guaranteed grain market with better price when they produce quality grain through the use of improved variety seeds.

Also grain with good taste is highly demanded for household use and food for diabetic people. People are increasingly getting awareness of the health benefit from consuming sorghum like prevention of cancer, reduce tumor incidence and lower blood pressure (Saleh et al., 2013). Sorghum is gluten free and good for diabetic control compared to other cereals, preventing cancer and many digestive problems (Rhodes, 2014). It also gives consumer a room to have multiple use of sorghum like biscuits, cakes, and breads. According to Dovi (2013) sorghum can be used to make biscuits because of its additional nutritional benefits in terms of minerals, dietary fibre and health promoting phytochemical. It is also preferred by most of brewing companies for opaque beer production since it meets taste and preferences of the consumers (Dabija et al.,2021). The use of improved sorghum variety resulted from market traits such as taste, brewing and cooking quality (Timu et al., 2014).

Difference in price between sorghum grain and improved sorghum variety has positive influence on farmer's decision to use improved sorghum variety. When the price of sorghum grain is higher compared to improved variety seeds, farmers will use more of improved sorghum variety in grain production because of the higher revenue that will be obtained from the market. Higher revenue will help farmers to cover production cost during grain production and will also motivate sorghum farmers to engage more in commercialization. With market demand drivers that will influence farmers to use improved varieties will also motivate farmers to participate in the market since they will be able to meet market demand. Therefore, farmers to have better benefit from their produce; they should be influenced by market demand. This means that what to produce are determined by consumers, how to produce is determined by producers (Economics Online, 2019).

### 5.2 Factors affecting market participation among small-scale sorghum farmers

Probit model reveals that age has the negative effect on farmer's decisions to participate in the market. This means that the younger the farmers, the more likely they are to participate in market because they are more dynamic to change their agricultural practices. This concurs with Olwande and Mathenge (2011) & Moono (2015), that young, energetic, and active member of the household are more likely to participate in market. On the other hand, older farmers are risk averse and slow to adopt changes and technologies (Alene et al., 2008; Heltberg and Tarp, 2002).

The results also revealed the positive relationship between farm size and farmers' market participation. As farm size increases, the probability for market participation increases (Masoku et al., 2001: Abayneh and Tefera, 2013). In fact, the increase in farm size enhances the availability of the produce surplus for the market (Martey et al., 2012). The presence of large farm size enables farmers to allocate part of land for food crop production and part for cash crop production giving them the position to participate in the market. Moreover, increase in farm size boost total production thus sales of surplus produce (Hossain and Osmani, 2015). Govereh and Yayne (1999) found that the increase in land size pulled farmer's orientation towards diversification into cash cropping in Zimbabwe.

The model also displays the importance of farmer belonging to farmers' organization. Membership of farmer's organization increases farmer's access to production and marketing information and improves farmer's decision on market participation; it allows producers to reach economies of scale (Olwande and Mathenge, 2012) since farmers can market together and reduce transaction cost and other costs. It is also advantageous to farmers when engaging in group marketing as well as provision of credit among group members. Being in farmers' organization is usually used as a proxy to farmers' social capital and other benefit including information resources, reciprocal labor-hire management and consequently improve farmer's decision making (Deininger and Feder, 2009). Guaranteed market influence farmers to use improved sorghum variety since it increases sorghum production and reduce extra cost for market search (Alemaw, 2014).

### 5.3 Comparative advantage of using improved sorghum varieties

Users of improved sorghum varieties obtained high gross margin compared to non-users probably due to high yield and high average price obtained as the result of using improved sorghum variety. Users were offered high price since they meet market demand in terms of quality and quantity and that gave them a promising market for next season, in terms of affordability of improved sorghum variety and guaranteed market of their grain. Improved sorghum variety used by farmers in Mali also showed average positive gross return, implying that farmers investment in the use of sorghum improved variety seeds is profitable (Miklyaev et al., 2017). Because of the use of improved sorghum variety, users had high production cost from agronomic practices so as compliment the improved variety seeds for high quality and quantity grain. In case of non-users who faced high seed costs because they used more seeds than the required seed rate (7–10 kg/ha) (Africa Soil Health Consortium, 2014), this is due to non-user farmers broadcasting seeds as planting method but since they hardly follow agronomic advice. Similar study done by Obayelu and Ajayi (2018) who noted that non-user of improved variety incurred high seed cost due to seed broadcasting. Non-users believed that it is easy to maintain unimproved sorghum variety compared to improved sorghum variety.

Because of low quality sorghum grain obtained by non-users, grading and packaging was costly and hardly marketable. Low price offered to their sorghum grain gives less ability to acquire improved variety seeds for the next season. In order to encourage farmers to use improved variety seeds, they need to know and understand market demand and access to seeds (Wangari, 2013) i.e., grain market pulls seed market. Based on gross margin from users and non-users, we reject the null hypothesis that users and non-users had no significant difference in gross margins. There is significant different in farmer's gross margin per ha for using improved sorghum variety between users and non-users.

### 5.4 Other sorghum attributes (product type) that led to the use of improved sorghum varieties

Apart from market demand factors, sorghum farmers consider other attributes such as color of the grain, use of sorghum product type and quality and price of grain before adopting the use improved seeds (FEWS NET, 2018). Majority of sorghum grain in Tanzania is used for consumption, where its commonly consumed as whole grain or processed flour (Wambungu, 2011) and as source of raw material in brewing industry (Food Security Deparment, 2004). In Eastern and Southern part of Africa, sorghum is mostly used as a source of raw material in brewing industry for example, Togo uses about 60% of national sorghum production to produce sorghum beer (Dabija et al.,2021). White and red sorghum are most common incorporated into opaque beer as malt (Dabija et al.,2021) but white sorghum is highly preferred for opaque beer and beer powder (Msangula, 1993) due to its low tanning composition (FEWS NET, 2018). Improved sorghum varieties such as NACO Mtama 1 and Macia are also highly preferred by brewing industry due to their attractive color and palatability (Aloyce et al.,2017). In Tanzania brewing companies use both white and red sorghum in production of clear beer (Roh, 2012). Nigeria and Rwanda use white sorghum for starch and malt in brewing companies (Rohrbach et al.,1992). For food, white sorghum is highly preferred due to high palatability and red sorghum for its nutrients that are good for diabetic people (FEWS NET, 2018). it's obviously that the use of improved variety can also be influenced by other attributes i.e., color of the grain and sorghum product type and quality since it is expected that the demand from sorghum grain for example in brewing industry will increase (American sorghum, 2016). Therefore, seed breeders should consider all traits when developing new improved variety in order to meet market demand (Aloyce et al.,2017).

## 6 Conclusion

When farmers produce sorghum grain based on market demand, it guarantees farmer's better grain price of their grain and drives farmers to use improved variety seeds, where taste and preference and price difference between grain and seeds have positive relationship with the use of improved variety seeds. It's advantageous for farmers to use improved varieties even under low input, compared to landraces, it assures farmers and grain off-takers quality grain and meet market demand. Probit analysis showed that factors such as sex, group membership, number of years in school and farm size affect positively market participation among small-scale sorghum farmers, while age had negative relationship with farmer's market participation. Younger farmers are more likely to adopt changes and technologies such as the use improved variety seeds. Market participation is a foundation and an outcome of economic development. It promotes the linkage between the input and output agricultural markets. Increase in farmers' market participation stimulates the use of improved variety seeds. Furthermore, in consideration of sorghum attributes (product type) with market demand factor, farmers better produce what is needed in the market based on the use of the attributes preferred and that will give farmers more reason to use a specific type of improved variety. Increase in improved sorghum variety accessibility and affordability could play a vital role in increasing awareness, knowledge and use of improved variety seeds and also provide a room for investment opportunities for seed producers, farmers and grain off-takers. Therefore, there is a need to reinforce agricultural services to play a more prominent role in delivering information, improved variety seed and training services to farmers. Communication between farmers, grain off-takers and agricultural and market actors should be much clear and effective, so that farmers could have market and agronomy information.

Also, stakeholders should emphasize on producing/develop sorghum varieties that are not only have good characteristic in farm level but also in market level accompanied with preferred attributes so as to meet market demand.

## Declarations

### Author contribution statement

Elizabeth Kalema: Conceived and designed the experiments; Performed the experiments; Analyzed and interpreted the data; Contributed reagents, materials, analysis tools or data; Wrote the paper.

Essegbemon Akpo: Conceived and designed the experiments; Performed the experiments; Analyzed and interpreted the data; Contributed reagents, materials, analysis tools or data.

Geoffrey Muricho; Chris O. Ojiewo; Rajeev K. Varshney: Conceived and designed the experiments; Contributed reagents, materials, analysis tools or data.

Justin Ringo: Contributed reagents, materials, analysis tools or data.

### Funding statement

This work was supported by ICRISAT as well as good collaboration form TARI-Hombolo and TARI-Naliendele through AVISA and HOPE projects funded by the 10.13039/100000865Bill and Melinda Gates Foundation, grant Number OPP1198373.

### Data availability statement

Data will be made available on request.

### Declaration of interests statement

The authors declare no conflict of interest.

### Additional information

No additional information is available for this paper.
